# Using Information and Communication Technologies for Family Communication and Its Association With Family Well-Being in Hong Kong: FAMILY Project

**DOI:** 10.2196/jmir.4722

**Published:** 2015-08-24

**Authors:** Man Ping Wang, Joanna TW Chu, Kasisomayajula Viswanath, Alice Wan, Tai Hing Lam, Sophia S Chan

**Affiliations:** ^1^ The University of Hong Kong School of Nursing Hong Kong China (Hong Kong); ^2^ The University of Hong Kong School of Public Health Hong Kong China (Hong Kong); ^3^ Harvard TH Chan School of Public Health Center for Community-Based Research, Dana-Farber Cancer Institute/Department of Social and Behavioral Sciences Cambridge, MA United States

**Keywords:** information and communication technologies, family well-being, family communication, Chinese

## Abstract

**Background:**

Family communication is central to the family and its functioning. It is a mutual process in which family members create, share, and regulate meaning. Advancement and proliferation of information and communication technologies (ICTs) continues to change methods of family communication. However, little is known about the use of different methods for family communication and the influence on family well-being.

**Objective:**

We investigated the sociodemographic factors associated with different methods of family communication and how they are associated with perceived family harmony, happiness, and health (3Hs) among Chinese adults in Hong Kong.

**Methods:**

Data came from a territory-wide probability-based telephone survey using the Family and Health Information Trend survey (FHInTs). Frequency of family communication using different methods (ie, face-to-face, phone, instant messaging [IM], social media sites, and email) were recoded and classified as frequent (always/sometimes) and nonfrequent (seldom/never) use. Family well-being was measured using 3 questions of perceived family harmony, happiness, and health with higher scores indicating better family well-being. Adjusted odds ratios for family communication methods by sociodemographic characteristics and adjusted beta coefficients for family well-being by communication methods were calculated.

**Results:**

A total of 1502 adults were surveyed. Face-to-face (94.85%, 1408/1484) was the most frequent means of communication followed by phone (78.08%, 796/1484), IM (53.64%, 796/1484), social media sites (17.60%, 261/1484), and email (13.39%, 198/1484). Younger age was associated with the use of phone, IM, and social media sites for family communication. Higher educational attainment was associated with more frequent use of all modes of communication, whereas higher family income was only significantly associated with more frequent use of IM and email (*P*=.001). Face-to-face (beta 0.65, 95% CI 0.33-0.97) and phone use (beta 0.20, 95% CI 0.02-0.38) for family communication were associated with significantly higher levels of perceived family well-being.

**Conclusions:**

Socioeconomic disparities in using these information and communication technologies (ICT) methods for family communication were observed. Although traditional methods remain as the main platform for family communication and were associated with better family well-being, a notable proportion of respondents are using new ICT methods, which were not associated with perceived family well-being. Because ICTs will continue to diversify modes of family communication, more research is needed to understand the impact of ICTs on family communication and well-being.

## Introduction

Family communication through both verbal and nonverbal interactions plays a central role in maintaining family relationships and enhancing family well-being [1]. It provides the foundation for family members (individuals who are related through biological, marital, cohabitation, and/or emotional bonding) to share meaning, to be connected, to be flexible in changing family rules, to achieve satisfaction, and to express and share attitudes, values, and beliefs [2,3]. Family communication includes the content (both verbal and nonverbal), frequency, and nature of family interactions, which defines the family and constructs family relationships [1]. Although a Western perspective often defines communication as an expression of “self” [4,5], a Chinese perspective defines communication as a way to develop and maintain personal relations and to reaffirm their membership in their respective social networks [6,7]. Nonconfrontational communication (harmony) is valued in Chinese culture and refers to expressing one’s thoughts and feelings in an indirect and implicit manner [7]. Such pattern of communication is not only to preserve an individual’s dignity, but also to protect family harmony and ties [7].

Family communication comprises an important part of several Western theories and models (eg, Family System Theory, Social Learning Theory, Olson’s Circumplex Model, and McMaster Model of Family Functioning). Olson’s Circumplex model [1] and the McMaster Model of Family Functioning [8] posit that a well-functioning family is characterized by positive communication which provides a basis for higher level of family cohesion and adaptability. Family communication has also been argued to be vital for family harmony, happiness, and health that underlie family well-being from a Chinese perspective [9,10].

In addition to the traditional means of communication, such as face-to-face and phone, new forms of information and communication technologies (ICTs), such as instant messaging (IM), social media sites (eg, Facebook, Twitter), and email, allow individuals to communicate and interact with one another [11,12]. Each medium has unique attributes and provides benefits otherwise not available from other means of communication. Face-to-face communication possesses nonverbal elements, instant feedback, complete identification, and real-time interaction [13]. The phone, although it lacks visual communication cues, provides instant feedback and real-time interaction across a wide geographical range. IM and email allow for words (and now photographs, videos, and audio clips) to be asynchronously exchanged among individuals and family groups. Social media sites allow for interconnectivity and provide an avenue for texts, photos, and video sharing [14]. ICTs also create a new pattern of family communication [12]; individuals are now able to perform multiple media tasks at the same time and interact with multiple individuals simultaneously. Compared with traditional forms of communication, ICTs are able to break barriers of time, space, location, and distance in making virtual communication accessible, feasible, and efficient [14,15]. Indeed, the rapid development of ICTs has changed and continues to transform the ways in which families interact and communicate [14,15].

Although a growing number of studies have examined communication behaviors (eg, pattern, frequency, and usage of ICTs) and interpersonal relationships and family functioning [12,14,15], it is uncertain whether ICTs enhance or weaken family relationships [14,15]. ICT use may increase the time families spend together, strengthen family bonds, improve family communication, and enable the maintenance of family relationships [16-20]. Other studies have suggested that quality family time has been significantly reduced and overuse of ICTs can lead to isolation from the family and failure to develop normal modes of expression, affecting the quality of family relationships [18,21-23].

Hong Kong, the most Westernized and urbanized city in China, is one of the most technologically advanced and connected cities in the world with ICTs readily integrated into the daily lives of Chinese people. Most households (78%) have personal computers at home connected to the Internet [24]. Fixed-line residential phone penetration exceeds 100% and mobile phone (mostly with multiple ICT functions) subscription plans penetrate more than 230% [24]. Therefore, Hong Kong provides an appropriate platform to understand how different modes of communication are used and the influence it has on family well-being. This study examines the use of ICTs for family communication and their influence on perceived family well-being.

## Methods

### Sampling

As part of the FAMILY Project, the Hong Kong Family and Health Information Trends Survey (FHInTs) was conducted from August 2012 to October 2012 using probability-based telephone surveys to collect information on general public opinions and behaviors on family health, information use, and health communication. Details of the survey design have been reported elsewhere [25,26]. In brief, a 2-stage random sampling method was used. First, telephone numbers were retrieved from residential telephone directories that covered approximately 76% of Hong Kong residents [24]. A computer program was used to generate a list of the telephone numbers in random order for interview. Invalid household numbers, nonresponses, and ineligible households (people aged <18 years or not able to speak Cantonese) were excluded. Second, after initial introduction of the study purpose by the interviewers, adult respondents were asked how many eligible individuals were living in the household. All eligible individuals were listed and the individual with the next birthday closest to the interview day was elected for interview. Each interview took approximately 25 minutes to complete. A total of 2127 individuals were eligible; of those, 1502 adults were successfully interviewed yielding a response rate of 70.62%. Ethics approval was granted by the Institutional Review Board (IRB) of the University of Hong Kong/Hospital Authority Hong Kong West Cluster. Verbal informed consents were obtained and recoded verbatim, and the procedure was approved by the IRB.

### Measurements

The definition of families (family members who are related through biological, marital, cohabitation, and/or emotional bonding) was explained to the respondents before asking questions about family communication and family well-being. The prevalence of different methods of communication was assessed by asking respondents how often each method (ie, face-to-face, phone, IM, social media sites, and email) was used to communicate with family with responses of “very often,” “sometimes,” “seldom,” and “never.” Perceived family harmony, happiness, and health (3Hs) are regarded as the main component of family well-being in Chinese society [9,10]. Family 3Hs were assessed by using 3 separate questions asking respondents to give a score from 0-10. Family well-being was calculated based on the composite score of the 3Hs with higher scores indicating better family well-being (possible total score ranged from 0-30). The test-retest reliability in another sample yielded an alpha=.81, showing that the scale was reliable over 1 month. The internal consistency of the scale was alpha=.84 for this sample. Socioeconomic status (SES) was measured using educational attainment, employment status, and monthly household income. Educational attainment was categorized as primary or below, secondary, and tertiary or above. Employment status was categorized as full-time, part-time, self-employed, and unemployed. Monthly household income was categorized as ≤HK $9999, HK $10,000-$19,999, HK $20,000-$29,999, HK $30,000-$39,999, HK $40,000-$59,999, and ≥HK $60,000 (US $1=HK $7.8).

### Statistical Analysis

All data were weighted by sex and age using Hong Kong 2013 census data. Descriptive statistics were used to report prevalence of different methods of family communication. Associations of different methods to communicate with family by sex, age, marital status, and SES indicators (ie, income and educational attainment) were assessed by logistic regression, which yielded adjusted odds ratios (AORs) of family communication methods. Associations between perceived Family 3Hs (harmony, happiness, and health), family well-being, and different family communication methods were analyzed in a separate binary logistic regression model adjusting for sociodemographic characteristics. All analyses were conducted with SPSS 20 (SPSS Inc, Chicago, IL, USA).

## Results

Of 1502 respondents, 54.51% (819/1502) were female, 73.39% (1103/ 1500) were aged between 25 and 64 years, and 63.33% (950/1500) were married or cohabitating (Table 1). Most respondents had secondary or greater education (85.60%, 1286/1502) and 63.24% (822/1300) had monthly family income of HK $20,000 or greater (average monthly income in Hong Kong was HK $20,200). Among 611 unemployed participants (40.66%, 611/1502), 45.9% (281/611) were retired and 35.1% (215/611) were homemakers.

**Table 1:** Sociodemographic characteristics of sample (N=1502).

Demographics		Unweighted, n (%)	Weighted, %
**Sex**			
	Male	573 (38.15)	45.59
	Female	929 (61.85)	54.51
**Age** ^a^			
	18-24	126 (8.40)	9.94
	25-34	146 (9.73)	17.76
	35-44	153 (10.20)	18.55
	45-54	312 (20.80)	20.60
	55-64	387 (25.80)	16.49
	≥65	376 (25.07)	16.67
**Marital status** ^b^			
	Single	328 (21.88)	30.57
	Married/cohabitating	1038 (69.25)	63.33
	Other (divorced/widowed)	133 (8.87)	6.09
**Educational attainment**			
	Primary or below	318 (21.17)	14.40
	Secondary	733 (48.80)	47.68
	Tertiary or above	451 (30.03)	37.92
**Employment status**			
	Full-time	548 (36.48)	47.49
	Part-time	135 (8.99)	8.77
	Self-employed	38 (2.53)	3.09
	Unemployed	781 (52.00)	40.66
**Monthly household income (HK $)** ^c^			
	≤9999	302 (23.65)	17.25
	10,000-19,999	269 (21.06)	19.51
	20,000-29,999	214 (16.76)	18.31
	30,000-39,999	172 (13.47)	15.18
	40,000-59,999	168 (13.16)	15.48
	≥60,000	152 (11.90)	14.27

^a^ Missing (unweighted=2, weighted=2).

^b^ Missing (unweighted=3, weighted=2).

^c^ Missing (unweighted=225, weighted=202).

The most frequent means of communication was through face-to-face (94.85%, 1408/1484) followed by telephone (78.08%, 1159/1484) and IM (53.64%, 796/1484) (Table 2). Some have also used social media sites (17.60%, 261/1484) and email (13.39%, 198/1484) for family communication. Although 63.18% (938/1484) of respondents reported never using email (followed by social media sites 59.65%, 885/1484), only a few (0.85%, 13/1484) reported never having communicated with family face-to-face.

**Table 2:** Prevalence (weighted) of different methods to communicate with family (N=1484).

Means of communication	Prevalence, n (%)			
	Very often	Sometimes	Seldom	Never
Face-to-face	1199 (80.77)	209 (14.08)	64 (4.30)	13 (0.85)
Mobile phone/phone	637 (42.91)	522 (35.18)	219 (14.74)	107 (7.18)
Instant messaging instruments (eg, WhatsApp, WeChat [WeiXin], LINE)	458 (30.84)	338 (22.80)	183 (12.31)	505 (34.05)
Social media sites (eg, Facebook, Twitter, Google+, WeiBo)	95 (6.43)	166 (11.17)	338 (22.75)	885 (59.65)
Email	53 (3.59)	145 (9.80)	348 (23.44)	938 (63.18)

Compared with males, females used IM more frequently (AOR 1.56, 95% CI 1.19-2.03) (Table 3). Younger age was associated with more frequent use of phone (*P*=.001), IM (*P*<.001), and social media sites (*P*<.001). Higher adjusted odds ratio of email use was observed for older age group (*P*=.03), particularly for respondents’ aged 55 to 64 years of age (AOR 3.83, 95% CI 1.21-12.09). Compared with respondents with low education level (ie, primary or less), respondents with higher education had more frequent use of all different modes of communication. In particular, respondents with tertiary or greater level of education were strongly associated with IM (AOR 3.39, 95% CI 2.00-5.77) and email use (AOR 4.52, 95% CI 2.12-9.66). Higher household income was associated with more frequent IM (*P*<.001) and email use (*P*=.001). No association was observed for employment status with communication models.

**Table 3:** Associations of sociodemographic characteristics and use of different methods to communicate with family.^a^

Demographics		Face-to-face		Phone		Instant messaging		Social media sites		Email	
		AOR (95% CI)	*P*	AOR (95% CI)	*P*	AOR (95% CI)	*P*	AOR (95% CI)	*P*	AOR (95% CI)	*P*
**Sex**											
	Male	1		1		1		1		1	
	Female	0.68 (0.39, 1.20)	.19	1.29 (0.96, 1.73)	.10	1.56 (1.19, 2.03)	.001	1.11 (0.81, 1.51)	.52	1.28 (0.88, 1.87)	.19
**Age (years)**			.02^b^		.001^b^		<.001^b^		<.001^b^		.03^b^
	18-24	1		1		1		1		1	
	25-34	1.50 (0.45, 5.02)	.51	0.89 (0.46, 1.71)	.73	0.65 (0.39, 1.10)	.11	1.50 (0.84, 2.68)	.17	1.42 (0.49, 4.13)	.52
	35-44	1.26 (0.34, 4.64)	.73	0.48 (0.24, 0.96)	.04	0.61 (0.34, 1.08)	.09	0.98 (0.51, 1.88)	.94	1.51 (0.49, 4.69)	.48
	45-54	0.73 (0.20, 2.70)	.64	0.42 (0.21, 0.87)	.02	0.39 (0.21, 0.71)	.002	0.66 (0.33, 1.35)	.26	2.81 (0.91, 8.66)	.07
	55-64	0.61 (0.16, 2.29)	.46	0.32 (0.15, 0.67)	.003	0.28 (0.15, 0.53)	<.001	0.62 (0.29, 1.32)	.21	3.83 (1.21, 12.09)	.02
	≥65	0.44 (0.11, 1.71)	.24	0.30 (0.14, 0.65)	.002	0.09 (0.04, 0.18)	<.001	0.29 (0.12, 0.73)	.009	1.74 (0.49, 6.12)	.39
**Marital status**											
	Single	1		1		1		1		1	
	Married/cohabitating	2.83 (1.17, 6.86)	.02	1.68 (1.05, 2.68)	.03	1.94 (1.28, 2.93)	.002	1.03 (0.66, 1.61)	.90	3.52 (1.86, 6.65)	<.001
	Others	0.93 (0.32, 2.68)	.90	2.28 (1.08, 4.83)	.03	1.38 (0.67, 2.82)	.38	0.63 (0.23, 1.70)	.36	2.24 (0.75, 6.67)	.15
**Education**			.005^b^		.03^b^		<.001^b^		.43^b^		<.001^b^
	Primary or below	1		1		1		1		1	
	Secondary	3.54 (1.80, 6.97)	<.001	2.04 (1.35, 3.09)	.001	3.03 (1.90, 4.84)	<.001	1.60 (0.84, 3.04)	.15	1.78 (0.89, 3.57)	.11
	Tertiary or above	3.21 (1.32, 7.78)	.01	1.84 (1.12, 3.04)	.02	3.39 (2.00, 5.77)	<.001	1.12 (0.55, 2.27)	.76	4.52 (2.12, 9.66)	<.001
**Employment**											
	Full-time	1		1		1		1		1	
	Part-time	1.00 (0.36, 2.81)	.99	0.73 (0.43, 1.26)	.26	0.88 (0.55, 1.42)	.60	0.86 (0.47, 1.57)	.63	1.02 (0.52, 2.03)	.95
	Self-employed	0.71 (0.13, 4.06)	.70	0.71 (0.34, 1.52)	.38	0.98 (0.48, 2.00)	.95	1.07 (0.48, 2.39)	.88	1.04 (0.45, 2.41)	.93
	Unemployed	0.96 (0.46, 2.03)	.92	0.82 (0.56, 1.21)	.32	0.79 (0.57, 1.11)	.18	0.94 (0.63, 1.41)	.76	0.87 (0.55, 1.39)	.57
**Family income (HK $)**			.53^b^		.38^b^		<.001^b^		.02^b^		.001^b^
	≤9999	1		1		1		1		1	
	10,000-19,999	1.24 (0.58, 2.67)	.58	1.24 (0.77, 2.01)	.38	1.28 (0.80, 2.03)	.31	0.86 (0.46, 1.61)	.64	1.83 (0.85, 3.95)	.12
	20,000-29,999	2.13 (0.79, 5.75)	.14	0.67 (0.41, 1.10)	.11	1.48 (0.91, 2.40)	.11	0.95 (0.50, 1.80)	.89	1.76 (0.79, 3.91)	.16
	30,000-39,999	2.61 (0.82, 8.32)	.10	0.94 (0.55, 1.62)	.82	2.29 (1.37, 3.82)	.002	1.14 (0.59, 2.19)	.70	1.81 (0.80, 4.13)	.16
	40,000-59,999	0.85 (0.33, 2.14)	.73	1.31 (0.74, 2.32)	.36	2.84 (1.68, 4.81)	<.001	1.64 (0.86, 3.13)	.13	2.71 (1.22, 5.99)	.01
	≥60,000	2.68 (0.67, 10.60)	.16	1.43 (0.77, 2.68)	.26	3.63 (2.05, 6.40)	<.001	1.39 (0.70, 2.76)	.35	2.94 (1.31, 6.59)	.009

^a^All variables were mutually adjusted. Frequency of use of communication methods was dichotomized as “very often/sometimes” and “seldom/never.”

^b^ *P* value

Frequent use of face-to-face communication was strongly associated with perceived family harmony (AOR 0.82, 95% CI 0.45-1.19), family happiness (AOR 0.59, 95% CI 0.22-0.96), family health (AOR 0.54, 95% CI 0.16-0.91), and overall family well-being (AOR 0.65, 95% CI 0.33-0.97) (Table 4). Similarly, more phone use was associated with family harmony (AOR 0.22, 95% CI 0.02-0.43), family happiness (AOR 0.36, 95% CI 0.15-0.56), and overall family well-being (AOR 0.20, 95% CI 0.02-0.38). Using new ICTs (ie, IM, social media sites, and email) was positively, but nonsignificantly, associated with family 3Hs and well-being.

**Table 4:** Association between family 3Hs and the use of different methods to communicate with family.^a^

Means of communication		Family harmony		Family happiness		Family health		Family well-being	
		Mean (SD)	Beta (95% CI)^b^	Mean (SD)	Beta (95% CI)^b^	Mean (SD)	Beta (95% CI)^b^	Mean (SD)	Beta (95% CI)^b^
**Face-to-face**									
	Seldom/never	7.04 (2.31)	0	6.76 (2.32)	0	6.87 (2.17)	0	6.89 (2.01)	0
	Always/sometimes	7.78 (1.48)	0.82 (0.45, 1.19)^e^	7.39 (1.52)	0.59 (0.22, 0.96)^d^	7.39 (1.50)	0.54 (0.16, 0.91)^d^	7.52 (1.29)	0.65 (0.33, 0.97)^e^
**Phone**									
	Seldom/never	7.58 (1.77)	0	7.08 (1.92)	0	7.27 (1.72)	0	7.32 (1.55)	0
	Always/sometimes	7.78 (1.46)	0.22 (0.02, 0.43)^c^	7.44 (1.45)	0.36 (0.15, 0.56)^d^	7.39 (1.49)	0.06 (–0.14, 0.27)	7.54 (1.27)	0.20 (0.02, 0.38)^c^
**Email**									
	Seldom/never	7.70 (1.55)	0	7.32 (1.61)	0	7.37 (1.56)	0	7.47 (1.36)	0
	Always/sometimes	7.98 (1.40)	0.14 (–0.10, 0.39)	7.62 (1.31)	0.11 (–0.15, 0.36)	7.35 (1.39)	–0.20 (–0.45, 0.05)	7.66 (1.18)	0.02 (–0.20, 0.23)
**Instant messaging**									
	Seldom/never	7.70 (1.67)	0	7.28 (1.76)	0	7.29 (1.66)	0	7.43 (1.46)	0
	Always/sometimes	7.77 (1.41)	0.01 (–0.17, 0.19)	7.43 (1.39)	0.06 (–0.13, 0.24)	7.43 (1.43)	–0.01 (–0.19, 0.17)	7.55 (1.22)	0.02 (–0.14, 0.17)
**Social media sites**									
	Seldom/never	7.72 (1.57)	0	7.36 (1.62)	0	7.37 (1.57)	0	7.49 (1.38)	0
	Always/sometimes	7.80 (1.35)	0.17 (–0.05, 0.38)	7.35 (1.32)	0.03 (–0.19, 0.24)	7.38 (1.36)	0.00 (–0.22, 0.21)	7.52 (1.11)	0.07 (–0.11, 0.26)

^a^Frequencies of use of communication methods were dichotomized as “very often/sometimes” and “seldom/never;” Family 3Hs ranged from 0 (totally unhealthy/unhappy/inharmonious) to 10 (very healthy/happy/harmonious), with 5 indicating “half-half.”

^b^ Adjusting for sex, age, education attainment, monthly household income, and marital status.

^c^ *P*<.05

^d^ *P*<.01

^e^ *P*<.001

## Discussion

Although research on the interplay between technological advancements and family functioning are needed [11,15,27] and increasingly reported, little consensus has been found on the impact of ICTs on family well-being. Findings on Chinese population are scarce and our study provides the first evidence on ICTs use and perceived family well-being among Chinese adults.

Overall, the findings are consistent with studies elsewhere [12,28,29] that showed traditional means of communication (ie, face-to-face and phone) were most frequently endorsed compared with emerging ICTs (ie, IM, social media, and email). Our findings on the associations between sociodemographic characteristics and use of different communication methods also revealed similar trends and characteristics to those in the literature [30,31]. Younger individuals would have grown up in a generation riddled with new technology and are, therefore, more likely to embrace ICTs in various forms. Higher education level and higher household income were associated with more frequent ICT use (ie, IM and email). One possible explanation is that individuals with higher education were more likely to be professional workers or employed in office settings that have greater access to computers and mobile phones that allowed for IM and email use. Similarly, those with higher household income may have greater resources (eg, financially) and accessibility (eg, Internet connection at home, work, and mobile subscription plans) to ICTs.

Of significance is that traditional methods of communication (face-to-face and phone) were strongly associated with higher levels of perceived family well-being. This finding is especially important given that recent studies suggested a transformative trend toward more frequent use of ICTs than traditional methods of communication, particularly in the younger age group [15,18,30,32]. Specifically, we found that face-to-face communication was significantly related to all 3 dimensions of family well-being (harmony, happiness, and health). Using the phone as a communication method was also associated with higher levels of family harmony, happiness, and family well-being. One explanation is that the quality of communication through face-to-face and phones are richer than those of ICTs and, thus, provide greater communication satisfaction [28]. Face-to-face communication conveys verbal, nonverbal, and social context cues simultaneously and provides immediate and synchronized feedback. These are all fundamental qualities to establishing human relationships that, in turn, affect family well-being [33]. Particularly in Chinese societies, where the style of communication is often indirect (messages are often implicit and the meanings are to be inferred from contextual cues), face-to-face communication represents an important means of communication. Although not directly examining family well-being, another study reported similar findings on ICT use and perceived adolescent well-being [34]. More frequent use of social media sites by college students to communicate with their parents was more strongly associated with self-reported loneliness. On the other hand, frequent phone communication was associated with more positive qualities in the parent-child relationship, including greater satisfaction, intimacy, support, and instrumental aid [34].

Given the importance of face-to-face communication, our findings support the notion that ICTs should not replace traditional methods of communication, but rather should be utilized as a supplement. Studies also found when ICTs were used as a substitute, the effects on interpersonal relationships were negative [35]. The absence of nonverbal cues and tacit knowledge makes communication difficult and hinders relationship formation, cohesion, and trust [36]. For example, the lack of social presence creates an environment in which individuals easily misinterpret emotions and/or make incorrect assumptions. However, when used as a complement to face-to-face communication, ICTs facilitated the maintenance of interpersonal relationships [35]. These studies along with our findings suggest the importance of ICTs supplementing traditional methods of communication on improving family well-being. Therefore, efforts to improve family communication and well-being need to focus on informing individuals about the importance of face-to-face communication and the opportunities and pitfalls that ICTs bring.

One of the limitations of this study is the broad categories used in assessing the different mediums of communication. For example, we did not differentiate between fixed-line residential phone and mobile phone. We also did not incorporate videoconference services, such as Skype and Jaber, which at the time were seldom used. Another limitation is that we did not assess the geographical distance between family members, which can have an influence on the choice of communication methods [17]. None-the-less, others found that compared with other modes of communication, face-to-face communication showed a strong positive relationship to frequency of contact after controlling for locality [12]. The cross-sectional design cannot be used to determine causality. The sampling method only covered residential telephone directories; therefore, households that used mobile phones only were excluded. Finally, data from individuals younger than 18 years were not collected. Given that children and adolescents are more deeply immersed in the digital world, examining their behaviors and pattern of use may provide a clearer picture to how ICTs impact family well-being across different life spans.

This study suggests several avenues for future research. The quality of communication has rarely been measured [15,37]. The context and content of the dialog may provide more insight as to the quality of the communication that is likely to influence family relationships and family well-being more strongly. Future studies are also needed to examine the diverse range of ICTs, from preference and pattern of use to its association and causality.

Although traditional methods remained as the main platform for communication within the family and were associated with better family well-being, a notable proportion were using new ICT methods (ie, IM, social media sites, and email). Socioeconomic disparities in using these ICT methods for family communication were observed. Because ICTs will continue to diversify modes of family communication, more research is needed to understand the impact of ICTs on family communication and well-being.

## Abbreviations

3H: perceived family harmony, happiness, and health
AOR: adjusted odds ratio
FHInT: Family and Health Information Trend
ICT: information and communication technologies
IM: instant messaging
